# LAV-BPIFB4 reverses progeria-associated cardiac aging by restoring diastolic function and reducing senescence

**DOI:** 10.7150/ijbs.129092

**Published:** 2026-01-29

**Authors:** Unbin Chae, Young-Ho Park, Taeho Kwon, Kyung-Sook Chung, Sun-Uk Kim

**Affiliations:** 1 Futuristic Animal Resource and Research Center, Korea Research Institute of Bioscience and Biotechnology (KRIBB), Cheongju, Chungbuk 28116, Republic of Korea.; 2 Advanced Bioconvergence Department, KRIBB School, Korea National University of Science and Technology (UST), Daejeon 34113, Republic of Korea.; 3 Center for Gene and Cell Therapy, Korea Research Institute of Bioscience and Biotechnology (KRIBB), Daejeon 34141, Republic of Korea

A recent study published in *Signal Transduction and Targeted Therapy* by Yan Qiu demonstrated that activation of a longevity gene can partially reverse cardiac damage associated with Hutchinson-Gilford progeria syndrome (HGPS) 1. HGPS is primarily caused by a recurrent point mutation (c.1824 C>T, often designated as G608G) in exon 11 of the LMNA gene, which occurs de novo in most patients 2, 3. This mutation causes abnormal alternative splicing, impairing the function of a nuclear membrane protein called mutant lamin A or progerin. Progerin accumulates in the cell nucleus, disrupting nucleocytoskeletal coupling and contributing to characteristic symptoms of HGPS, including clinical features resembling premature aging, skeletal deformities, and shortened lifespan. Among the various systemic manifestations, cardiovascular dysfunction, particularly progressive atherosclerosis and cardiac fibrosis, represents the leading cause of mortality in HGPS patients 4. Consequently, the cardiovascular system has been identified as a primary therapeutic target in progeria research. Over the past decade, extensive efforts have been devoted to improving cardiac outcomes in HGPS, ranging from conventional pharmacological strategies aimed at mitigating vascular pathology 5 to the recent development of CRISPR-based approaches designed to correct the underlying LMNA mutation and selectively eliminate progerin expression 6, 7. These advances underscore the growing focus on restoring cardiovascular integrity as a central objective of effective therapeutic intervention in HGPS.

Recent studies have introduced a promising paradigm for drug target discovery grounded in the genetics of human extreme longevity 8. By systematically analyzing the genomes of individuals with exceptional lifespans, researchers have uncovered rare variants within evolutionarily conserved, aging-related pathways. These variants have undergone rigorous functional validation in both cellular and animal models, ultimately establishing a framework for identifying genetic alterations as molecular targets for the development of therapeutics aimed at prolonging healthspan. A compelling example of this approach is the BPI Fold Containing Family B Member 4 (BPIFB4) gene, particularly the longevity-associated variant (LAV; Ile229Val/Asn281Thr/Leu488Phe/Ile494Thr), which has been identified in the homozygous state in supercentenarians, individuals living beyond 110 years of age in good health 9. Enhanced expression of LAV-BPIFB4 in senescent vascular cells and aged mice not only restores cardiac function and vascularization but also confers substantial anti-aging benefits, thereby mechanistically linking longevity genetics to translational therapeutic applications 10. Notably, HGPS cells exhibited markedly reduced endogenous BPIFB4 expression, likely attributable to progerin-mediated suppression. Building on these insights, the present study posits that restoring BPIFB4 function may effectively counteract progerin-induced toxicity and ameliorate the cardiovascular dysfunction characteristic of HGPS.

The authors conducted experiments using 26-week-old HGPS mice that exhibited growth retardation compared to age-matched control mice (Figure 1) 1. Three-dimensional echocardiographic examinations revealed that untreated HGPS mice spontaneously developed left ventricular diastolic dysfunction between 7 and 8 months of age, characterized by a decreased E/A ratio and an elevated E/E' ratio. Conversely, HGPS mice receiving a single intraperitoneal injection of AAV9-LAV-BPIFB4 showed significant recovery of left ventricular diastolic function within 2 months post-transduction, with 2.5-fold enhancement of cardiac BPIFB4 protein expression. Histological analyses demonstrated that AAV9-LAV-BPIFB4 specifically reduced perivascular fibrosis, but not interstitial fibrosis, increased the number of coronary arterioles, and enhanced vascular smooth muscle cell coverage. Two months post-transduction, aortic tissue harvested from 8-month-old progeria mice revealed no detectable atherosclerotic lesions, consistent with the early-stage nature of this HGPS model rather than a direct anti-atherosclerotic effect of LAV-BPIFB4 1. Transduction of LAV-BPIFB4 into HGPS patient-derived fibroblasts led to reduced senescence and fibrotic markers, including calponin-1 (CNN1), smooth muscle α-actin (ACTA2), epiregulin (EREG), Col4A5, elastin, and β-galactosidase, without affecting progerin protein accumulation 1. This was achieved through suppression of the p53-p21 axis and modulation of EGFR-mediated fibrotic signaling.

The novelty of this study lies in identifying LAV-BPIFB4 as a therapeutic modality mechanistically distinct from progerin-elimination approaches 1. Rather than directly targeting progerin for degradation, LAV-BPIFB4 functions as a cellular resilience activator, restoring nucleolar integrity and function encompassing ribosome biogenesis, ribonucleoprotein assembly, and ribosomal protein interactions 1. Through this nucleolar-centric mechanism, LAV-BPIFB4 attenuates progerin-induced nucleolar and nuclear stress and nuclear dysfunction, thereby preserving diastolic performance, reducing cardiac fibrosis, and mitigating age-related senescence.

Importantly, this mechanism complements progerin-suppression strategies by enhancing cellular resilience rather than directly reducing progerin levels. While gene-editing interventions correct LMNA mutations or lower progerin burden, LAV-BPIFB4 reinforces nucleolar integrity, ribosome biogenesis, and stress-response capacity 1. Together, these complementary mechanisms may provide a multi-layered cardioprotective strategy for stabilizing the vulnerable myocardial environments.

In summary, this groundbreaking study reveals that LAV-BPIFB4, a protective variant identified in supercentenarian genetics, reverses cardiac dysfunction in progeria through a resilience-enhancing mechanism rather than direct progerin elimination. By selectively mitigating perivascular fibrosis and fortifying cardiomyocyte stress tolerance while preserving progerin expression, LAV-BPIFB4 establishes a fundamentally new therapeutic paradigm. These findings underscore that the systematic interrogation of human exceptional longevity genetics reveals multiple protective alleles with significant translational potential, not only for rare genetic aging diseases but also for age-related cardiovascular pathologies in the general population.

## Figures and Tables

**Figure 1:**
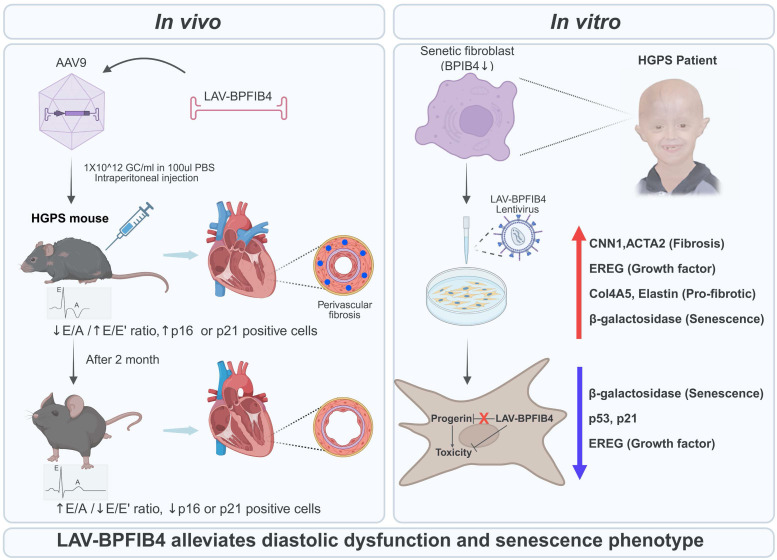
Therapeutic effect of LAV-BPIFB4 on HGPS-induced diastolic dysfunction and cellular senescence. Progeria mice without AAV-mediated LAV-BPIFB4 transduction developed left ventricular diastolic dysfunction, whereas mice treated with AAV9-LAV-BPIFB4 showed functional recovery 2 months post-transduction. Treatment significantly reduced perivascular fibrosis but did not affect interstitial fibrosis, increased coronary arteriole density and vascular smooth muscle cell coverage, while aortic examination revealed no detectable atherosclerotic lesions, consistent with the early-stage nature of this HGPS model. In patient-derived HGPS fibroblasts, LAV-BPIFB4 transduction downregulated senescence and fibrotic markers without altering progerin accumulation. These effects involved inhibition of the p53-p21 pathway and modulation of EGFR-mediated fibrosis. Figure created with BioRender.com.

## Abbreviations

HGPS: Hutchinson-Gilford progeria syndrome
LAV: Longevity-associated variant
CNN1: Calponin-1
ACTA2: Smooth muscle α-actin
EREG: Epiregulin
